# Genomic Characterization of 2 *Cutibacterium acnes* Isolates from a Surgical Site Infection Reveals Large Genomic Inversion

**DOI:** 10.20411/pai.v8i1.606

**Published:** 2023-10-05

**Authors:** D. Garrett Brown, Taylor A. Wahlig, Angela Ma, Laura K. Certain, Peter N. Chalmers, Mark A. Fisher, Daniel T. Leung

**Affiliations:** 1 Division of Infectious Diseases, University of Utah School of Medicine, Salt Lake City, Utah; 2 Department of Pathology, University of Utah School of Medicine, Salt Lake City, Utah; 3 ARUP Laboratories, Salt Lake City, Utah; 4 Department of Orthopaedic Surgery, University of Utah, Salt Lake City, Utah

**Keywords:** *Cutibacterium acnes*, surgical site infection, complete genome, genomic inversion

## Abstract

**Background:**

*Cutibacterium acnes* is a common commensal of human skin but may also present as an opportunistic pathogen in prosthetic joint and wound infections. Unfortunately, few complete genomes of *C. acnes* are publicly available, and even fewer are of isolates associated with infection. Here we report the isolation, characterization, and complete genomes of 2 *C. acnes* isolates from a surgical site infection of an elbow.

**Methods:**

We used standard microbiological methods for phenotypic characterization and performed whole genome sequencing on 2 *C. acnes* isolates using a combination of short-read and long-read sequencing.

**Results:**

Antibiotic susceptibility testing showed beta-lactamase negative and low minimal inhibitory concentrations to all antibiotics tested, with the exception of metronidazole. We assembled complete genomes of the 2 isolates, which are approximately 2.5 megabases in length. The isolates belong to the single-locus sequence type (SLST) H1 and the multi-locus sequence type (MLST) IB. Both isolates have similar composition of known virulence genes, and we found no evidence of plasmids but did find phage-associated genes. Notably, the 2 genomes are 99.97% identical but contain a large genomic inversion encompassing approximately half of the genome.

**Conclusions:**

This is the first characterization of this large-scale genomic inversion in nearly identical isolates from the same wound. This report adds to the limited numbers of publicly available infection-associated complete genomes of *C. acnes*.

## INTRODUCTION

Although *Cutibacterium acnes* is a well-known commensal of human skin, it can also present as an opportunistic pathogen [1]. Phylogenetic studies suggest that sequence phylotype may influence the pathogenic likelihood of specific strains [2, 3]. However, to date, only 34 complete *C. acnes* genomes are published on NCBI, and few of these were associated with infection. The additional sequencing of pathogen genomes can allow us to better use genomic information to infer clades or loci associated with pathogenicity. Here we report the complete genomes of 2 potentially pathogenic *C. acnes* isolates, collected from a surgical site infection of an elbow.

## METHODS

### Culturing and Growth

Samples collected from the irrigation and debridement procedures were submitted for anaerobic culture at the clinical microbiology laboratory. Upon arrival, samples were processed and plated onto Columbia sheep blood agar and pre-reduced Brucella, Bacteroides Bile Esculin, Laked-Kanamycin-Vancomycin, and Phenylethyl Alcohol agars. Columbia sheep blood plates were incubated in 5% CO_2_ conditions, whereas the remaining plates were incubated in anaerobic chambers. Following a 48-hour incubation, all plates were examined for bacterial growth. Small, white colonies growing on the anaerobic Brucella plates were subcultured onto Columbia sheep blood and Brucella agars for aerotolerance testing. Morphologically similar colonies only grew on the Brucella subcultures and were determined to be Gram-positive rods. MALDI-TOF mass spectrometry was used to confirm that all isolates that grew were *C. acnes*. Hemolysis was defined as any zone clearing observed on blood agar plates.

Out of the multiple *C. acnes* isolates recovered from the samples, antimicrobial susceptibility testing (AST) was performed on 1 representative pure culture of *C. acnes* isolate from the irrigation and debridement procedure. AST was performed by broth microdilution with supplemented Brucella broth with 5% lysed horse blood against the following agents: penicillin, amoxicillin/clavulanate, ampicillin/sulbactam, piperacillin/tazobactam, cefoxitin, ertapenem, imipenem, meropenem, clindamycin, moxifloxacin, and metronidazole. Minimum inhibitory concentration values without interpretations were reported for all agents due to the lack of broth microdilution interpretative breakpoints for non-*Bacteroides fragilis* group anaerobic bacteria according to Clinical Laboratory Standards Institute M100 guidelines. Absence of beta-lactamase activity was determined by the nitrocefin test.

### Isolate Sequencing

We selected 2 isolates for whole-genome sequencing. Colonies were isolated from plates, washed with cold 10% glycerol and pelleted. From the pellets, we extracted DNA using a Promega Wizard HMW DNA kit, following the gram-positive protocol with overnight incubation in lysozyme at 37°C. We performed both long-read Oxford Nanopore Technology (ONT) and short-read Illumina sequencing on purified DNA. For ONT sequencing, we prepared libraries following the NanoPore NBE_9065_V109_revJ_23May2018 protocol. Sequencing was performed on the MinION platform. Short-read sequencing was performed at the University of Colorado, using the Illumina NovaSeq6000 platform to generate 151 base-pair paired-end reads on libraries prepared using the Tecan UltraLow DNA library kit.

### Genome Assembly and Annotation

We removed adapters, barcodes, and demultiplexed MinION long read using the Guppy command guppy_barcoder, and options “-x auto -q 0 --trim_adapters --trim_barcodes”. To process the short reads, we used fastp to remove any detected adapters, low quality stretches of bases, and reads that were either low quality and/or less than 151 BPs (options “-y -D, --detect_adapters_for_pe and -l 151”) [4]. As our short-read sequencing was extensive, we randomly subset the reads to 20M total reads in each file using seqtk [5]. We then used the B-assembler genome assembler pipeline to assemble each genome; we used the hybrid mode, taking advantage of both our long and short reads. We then used the NCBI Prokaryotic Genome Annotation Pipeline (PGAP) to annotate our assembled genomes. We used Quast to compare our genome assemblies to the other publicly available *C. acnes* complete genomes, drawing on the *C. acnes* HL096PA1 genome as a reference.

### Comparative Genomic Analysis

We downloaded all publicly available *C. acnes* genomes on NCBI for genomic analysis. We used Phylogenetic and Molecular Evolution (PhaME) to build both FastTree and Randomized Axelerated Maximum Likelihood (RAxML) phylogenetic trees, using *C. acnes* HL096PA1, again, as the reference genome [6]. We used genomes with and without the annotation to build separate trees. We first ran PhaME on the chromosomal genomes on NCBI classified as complete or chromosomal (34 total genomes at time of analysis). We then used PhaME to build trees with all 426 genomes published on NCBI. Trees were visualized using the treeviewer at http://etetoolkit.org/treeview/ [7]. Multi-locus sequence type (MLST) was performed using the typer available at www.pubmlst.org/organisms/cutibacterium-acnes [8]. Single-locus sequence type (SLST) was performed using the typer available at www.medbac.dk/slst_server_script.html [9].

### Detection of Plasmids, Phages, and Virulence Factors

The output of B-assembler was examined to check for the presence of plasmids. We then ran PlasmidSeeker on our processed short reads to test for plasmid presence. We used PlasmidSeeker with the default settings and database or with 3 common *C. acnes* plasmids (CP003294.1 *Propionibacterium acnes* HL096PA1 plasmid; AP025555.1 *C. acnes* TP-CU411 plasmid pTP-CU411 DNA;NZ_CP012356.1 *C. acnes* strain PA_15_1_R1 plasmid unnamed). To confirm these findings, we built a Bowtie index using the *C. acnes* plasmids and used SAMtools to map the processed short reads from each genome against these plasmids [10, 11].

### PCR Validation of Genomic Inversion

Primers designed by Kasimatas et al were used to verify the genomic inversion in our isolates [12]. Following the previously described DNA isolation protocol, PCR was performed using Takara ExTaq, following the manufacturer's instructions. PCR was performed with the following cycling parameters: 94°C for 2 minutes, then 30 cycles of: 94°C for 10 seconds, 54°C for 1 minute, and 68°C for 7 minutes; then a final elongation step of 10 minutes at 68°C.

### Data Availability

All computational data and both bacterial isolates are available upon request. Data associated with this work are available in Bioproject PRJNA977060 and Biosamples SAMN35448566, SAMN35448567.

## RESULTS

### Isolation of Pathogenic *C. acnes*

An immunocompetent adult man presented with an elbow injury on his left (non-dominant) arm. After diagnosis of an acute extensor tendon tear with retraction, the patient underwent an open repair of the tendon at the lateral elbow without any immediate complications. One month post-operatively, he presented with swelling, induration, pain, and erythema of the surgical site. He also felt systemically ill with subjective fevers. The patient was prescribed cephalexin, and 2 days later, he underwent irrigation and debridement where significant gross purulence was encountered. All foreign material was removed, and 3 samples were taken for culture. While awaiting culture data, the patient started trimethoprim-sulfamethoxazole. All 3 cultures grew *C. acnes,* and the patient was subsequently switched to linezolid. Approximately a week later, irrigation and debridement were repeated due to continued signs of infection (erythema, pain, chills). Four more samples were taken for culture, 2 of which grew *C. acnes.* At this point, due to the unusual presentation of this case, isolates were saved from the 2 *C. acnes-*positive cultures from the second debridement. The patient was prescribed levofloxacin (which was stopped after *C. acnes* was the only organism to grow) and amoxicillin with clavulanate, which was continued for 1 month. Post-operatively, the patient recovered full range of motion and strength. At 2 years post-operatively, he remained free of infection and had full, painless function in the elbow.

### Phenotypic Characterization of *C. acnes* Isolates

**Figure 1.**
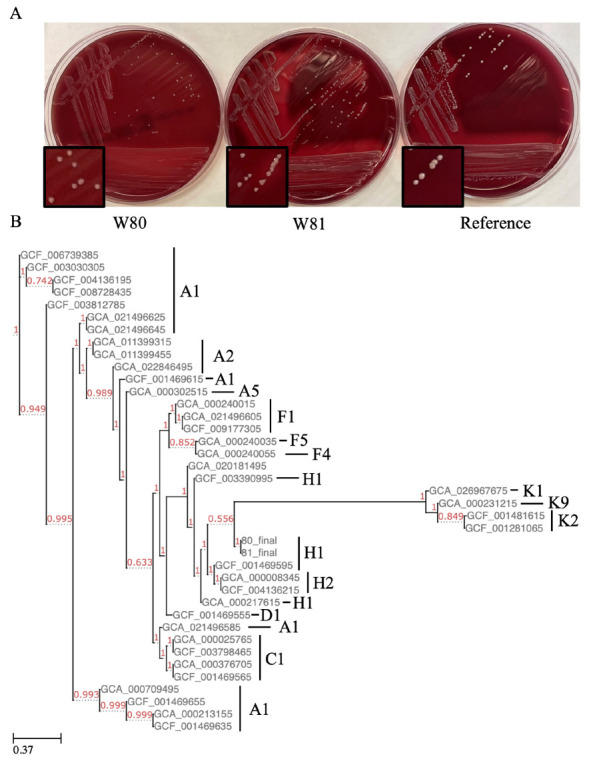
**Phenotype and phylogenetics of isolates.** (A) Colony morphology and hemolysis on blood agar plates. (B) FastTree assembled phylogenetic tree of all complete/chromosomal *C. acnes* genomes on NCBI. Red numbers are support values, and labels to the right represent SLST designation.

The *C. acnes* isolates were given the names W80 and W81. Isolate W80 underwent susceptibility testing: it was negative for beta-lactamase and had a low minimum inhibitory concentration to doxycycline and other tested antibiotics, suggesting susceptibility to antibiotics commonly used to treat *C. acnes* (Supplementary Table 1). This isolate, however, grew at all concentrations of metro-nidazole tested, a feature common to the species [13]. On blood agar, hemolysis was variable, with W80 showing no hemolysis and W81 displaying hemolysis (Figure 1A).

### Genomic Sequencing and Characterization

We performed short-read Illumina sequencing and long-read Oxford Nanopore sequencing on each isolate. We used the B-assembler pipeline to assemble the whole genomes [14]. For each isolate, we were able to assemble a 2.56 megabase pair (Mbp) circular genome (Table 1). Examination of each isolate revealed deep (91x and 103x) coverage and nearly 60% GC content, as expected. We performed quality control of genomes with QUAST 5.2.0, comparing results to the reference genome HL096PA1 [15]. When comparing to the reference strain, we found our assemblies to be similar in assembly characteristics to other complete genomes on NCBI. Using SLST, we classified the W80 isolate as type H1, and found that W81 was only 1 single-nucleotide polymorphism from this sequence type [9]. As expected, due to the H1 SLST designation, both isolates were classified as MLST group IB [2].

**Table 1.** Characteristics of Genome Assemblies and Annotations

Metric	W80	W81
Assembly Data		
Genome Size (BP)	2560519	2560653
Coverage (x)	91	103
GC Percent	60.01	59.94
Annotation Data		
Completeness	97.44	97.44
Contamination	0.62	0.62
Genes (total)	2,475	2,477
CDSs (total)	2,417	2,419
Genes (coding)	2,322	2,324
CDSs (with protein)	2,322	2,324
Genes (RNA)	58	58
rRNAs (5S, 16S, 23S)	3, 3, 3	3, 3, 3
complete rRNAs (5S, 16S, 23S)	3, 3, 3	3, 3, 3
tRNAs	45	45
ncRNAs	4	4
Pseudo Genes (total)	95	95
CDSs (without protein)	95	95
Pseudo Genes (ambiguous residues)	0 of 95	0 of 95
Pseudo Genes (frameshifted)	53 of 95	51 of 95
Pseudo Genes (incomplete)	25 of 95	28 of 95
Pseudo Genes (internal stop)	30 of 95	27 of 95
Pseudo Genes (multiple problems)	12 of 95	10 of 95
Sequence Typing		
Single-Locus (SLST)	H1	H1*
Multi-Locus (MLST)	IB	IB

* W81 is 1 single-nucleotide polymorphism from classification as H1.

We annotated our assembled genomes using PGAP. We identified 2475 and 2477 coding sequences in the W80 and W81 assemblies, respectively (Table 1). Identical numbers of RNA coding genes, rRNAs, tRNAs, ncRNAs and pseudogenes were observed in these isolates. We identified multiple loci from putative phages utilizing VirSorter2 [16]. Similar prophage sequences have been observed in the closely related KPA171202 strain [17]. In agreement with VirSorter2, PHAge Search Tool – Enhanced Release (PHASTER) identified an incomplete phage region, and PGAP annotation identified multiple phage-associated genes [18, 19].

We used PhaME to build phylogenetic trees composed of our isolates and the 34 complete/chromosomal genomes published on NCBI [6]. We found our assemblies clustered together with other IB (H1) phylotype strains (Figure 1B). This phylotype has previously been found to be enriched in infections [2, 3, 20]. We then built a tree with all the 400+ complete and incomplete genomes on NCBI. Interestingly, our isolates clustered with an incomplete genome of an isolate from a prosthetic joint infection from Madrid, Spain (Supplementary Figure 1A) [20]. Of note, this and other H1 isolates contain plasmids, but we could not detect any in our isolates when tested with B-assembler or PlasmidSeeker. To further confirm this, we mapped our processed short-read sequences against 3 plasmids identified in *C. acnes* genomes. One of these plasmids was previously reported in the nearby KPA171202 [21]. Further supporting the lack of plasmids detected by the other methods, we found each plasmid had a median coverage of 0 reads in each genome.

### Identification of Potential Virulence-Associated Loci

While characterization of virulence factors in *C. acnes* is limited, candidate loci are proposed. As performed by Cobian et al (2021), we used a curated table of 33 putative virulence factors identified in *C. acnes* strain KPA171202 to confirm the presence of suspected virulence factors in our isolates [1, 22]. All virulence factors were identified in our isolates, most with near 100% amino acid (AA) identity and coverage (Supplementary Figure 1B). However, DsA-2, a dermatan sulphate adhesin, was found in both isolates to span only 78% of KPA17202 reference. Interestingly, even though W81 appeared hemolytic, and W80 did not, all of these virulence factors were of similar length and AA composition in our 2 strains. Specifically, the verified hemolytic CAMP-2, and the 3 hemolysin genes were of 100% AA identity between W80 and W81[23]. This may suggest that variable expression of these genes may account for the phenotypic difference, as observed previously [17].

### Identification and Verification of Large-Scale Genomic Inversion

**Figure 2.**
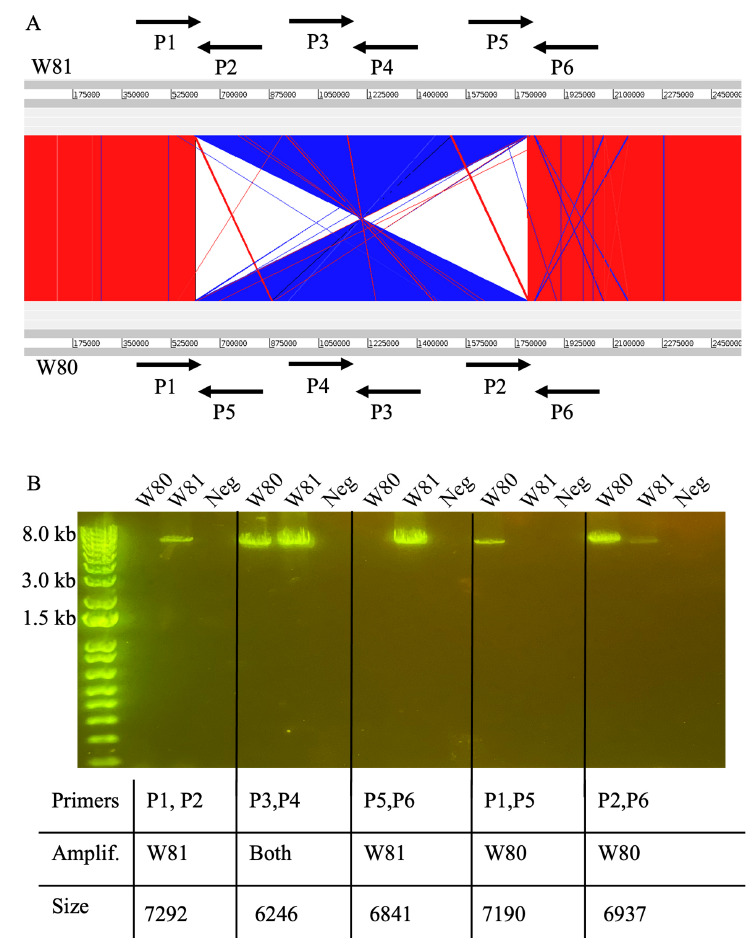
**Identification and verification of large genomic inversion between isolates**. (A) Comparison of W80 and W81 genomes, with blue illustrating inversion. Primers detailing verification strategy (designed by Kasimatas et al) shown with arrows. (B) PCR verification of genomic inversion.

Aligning the genomes of the 2 isolates revealed a large-scale genomic inversion (Figure 2A). Previous work identified the same inversion when comparing the strains HL096PA1 and KPA171202 [12]. This inversion was found to span ribosomal operons. Interestingly, the strains HL096PA1 and KPA171202 are of different phylogroups and sources, and our results represent the first identification of this large-scale inversion in nearly identical strains isolated from a single infection. As performed previously, we used a PCR method to validate the presence of the inversion. Figure 2A illustrates the scheme developed to verify orientation [12]. Figure 2B illustrates PCR results validating the genomic inversion.

## DISCUSSION

*C. acnes* may associate with humans in symbiotic relationships ranging from commensal to pathogenic. The number of complete *C. acnes* genomes available is limited, and addition of complete genomes of isolates from infections will help to answer future questions about its pathogenicity, with potential to inform infection prevention strategies and the development of vaccines and antimicrobial agents. Here we use short-read and long-read sequencing to characterize 2 pathogenic *C. acnes* isolates from a surgical site infection of the elbow of an immunocompetent adult. These isolates cluster with the phylotype (IB), as well as previously reported strains associated with infection.

We demonstrate that the strains had no evidence of plasmids, which in related strains, are known to carry additional virulence factors. The linear *C. acnes* plasmid pIMPLE-HL096PA1 carries clusters of adherence genes and adhesive pili that may promote pathogenicity [12]. The p15.1.R1 and related plasmids have been found in pathogenic strains, including the closest published strain to our isolates [20]. Our isolates seem to carry a strong pathogenic potential without these and other factors.

Although these isolates are nearly identical in sequence, including identical AA sequences in known hemolytic genes (CAMP and hemolysin genes), only W81 was hemolytic on blood agar. This may suggest that transcriptomic changes, or changes in genes not previously associated with hemolysis, are responsible for the variable phenotype. However, the change in hemolysis may be attributable to the large genomic inversion we identified in the 2 strains. A hemolytic phenotype in *C. acnes* is potentially associated with pathogenicity. Specifically, a report found an association between hemolysis and infection in patients receiving shoulder arthroplasty [24]. A study of isolates from prosthetic joints found a slight, albeit insignificant, association between hemolysis and infection in those of phylotype IB (the same as our 2 isolates) [3].

This inversion had only been previously described in 2 phylogenetically diverse strains [12], and our report is the first known identification of this inversion in 2 nearly identical isolates from the same infection. While the biological impact of this inversion in *C. acnes* has not been investigated, large genomic inversions in other bacterial organisms are associated with pathogenicity and persistent infection. For example, *Streptococcus pyogenes* may possess genomic inversions (albeit, asymmetrical inversions) that influence gene expression, growth, and virulence [25]. Another large-scale inversion, found in *Staphylococcus aureus,* is associated with the small colony variant phenotype [26]. Similar to our strains, the inversion is associated with persistent infection and decreased hemolysis. Interestingly, our isolate W81 is hemolytic and shares the same orientation with the hemolytic KPA171202 strain. However, no phenotypic testing was performed to assess biological impact of this inversion. The prevalence of this inversion is currently unknown, but of the complete *C. acnes* genomes on NCBI, only a small fraction (3 of 34) are of the KPA171202/W81 orientation. However, it should be noted that many of these genomes were constructed using solely short-read sequencing, and/or only used reference-based assembly, thus the presence of inversions in some samples may not have been detected.

## CONCLUSION

The availability of these genomes will increase our understanding of the pathogenicity of this organism. These isolates can be further studied to understand if *C. acnes* uses genomic inversion, as has been observed in other bacteria, to maintain persistent infection.

## SUPPLEMENTARY DATA

Supplementary materials are available at the *Pathogens and Immunity* website. Supplementary data may be provided by the authors to benefit the reader. Supplementary data are not copyedited and are the sole responsibility of the authors. Questions or comments related to supplementary materials should be addressed to the corresponding author.
