# Supplementary material for: Genomic Characterization of 2 Cutibacterium acnes Isolates from a Surgical Site Infection Reveals Large Genomic Inversion
Source: Pathog Immun. 2023 Oct 5;8(1):64–76. doi: 10.20411/pai.v8i1.606 (PMC10566467; doi:10.20411/pai.v8i1.606)
Supplement: Supplementary Table 1 [file pai-8-064-s01.pdf]

PATHOGENS AND IMMUNITY
IN ASSOCIATION WITH CASE WESTERN RESERVE UNIVERSITY AND THE RICHARD J. FASENMYER FOUNDATION

Supplementary Table 1. Minimum Inhibitory Concentration (MIC) for Select Antibiotics Against *C. acnes* strain W81.

**Antibiotic**	**MIC (µg/mL)**
penicillin	≤0.5
amoxicillin/clavulanate	≤0.12/0.06
ampicillin/sulbactam	≤0.25/0.12
piperacillin/tazobactam	≤1/4
cefoxitin	≤4
ertapenem	≤0.25
imipenem	≤0.12
meropenem	0.06
clindamycin	2
moxifloxacin	0.25
metronidazole	≥32

Brown DG, Wahlig TA, Ma A, Certain LK, Chalmers PN, Fisher MA, Leung DT. Genomic characterization of two *Cutibacterium acnes* isolates from a surgical site infection reveals large genomic inversion. *Pathogens and Immunity*. 2023;8(1):64-76. doi: 10.20411/pai.v8i1.606
