# Supplementary material for: Genomic Characterization of 2 Cutibacterium acnes Isolates from a Surgical Site Infection Reveals Large Genomic Inversion
Source: Pathog Immun. 2023 Oct 5;8(1):64–76. doi: 10.20411/pai.v8i1.606 (PMC10566467; doi:10.20411/pai.v8i1.606)
Supplement: Supplementary Figure 1 [file pai-8-064-s02.pdf]

PATHOGENS AND IMMUNITY
IN ASSOCIATION WITH CASE WESTERN RESERVE UNIVERSITY AND THE RICHARD J. FASENMYER FOUNDATION

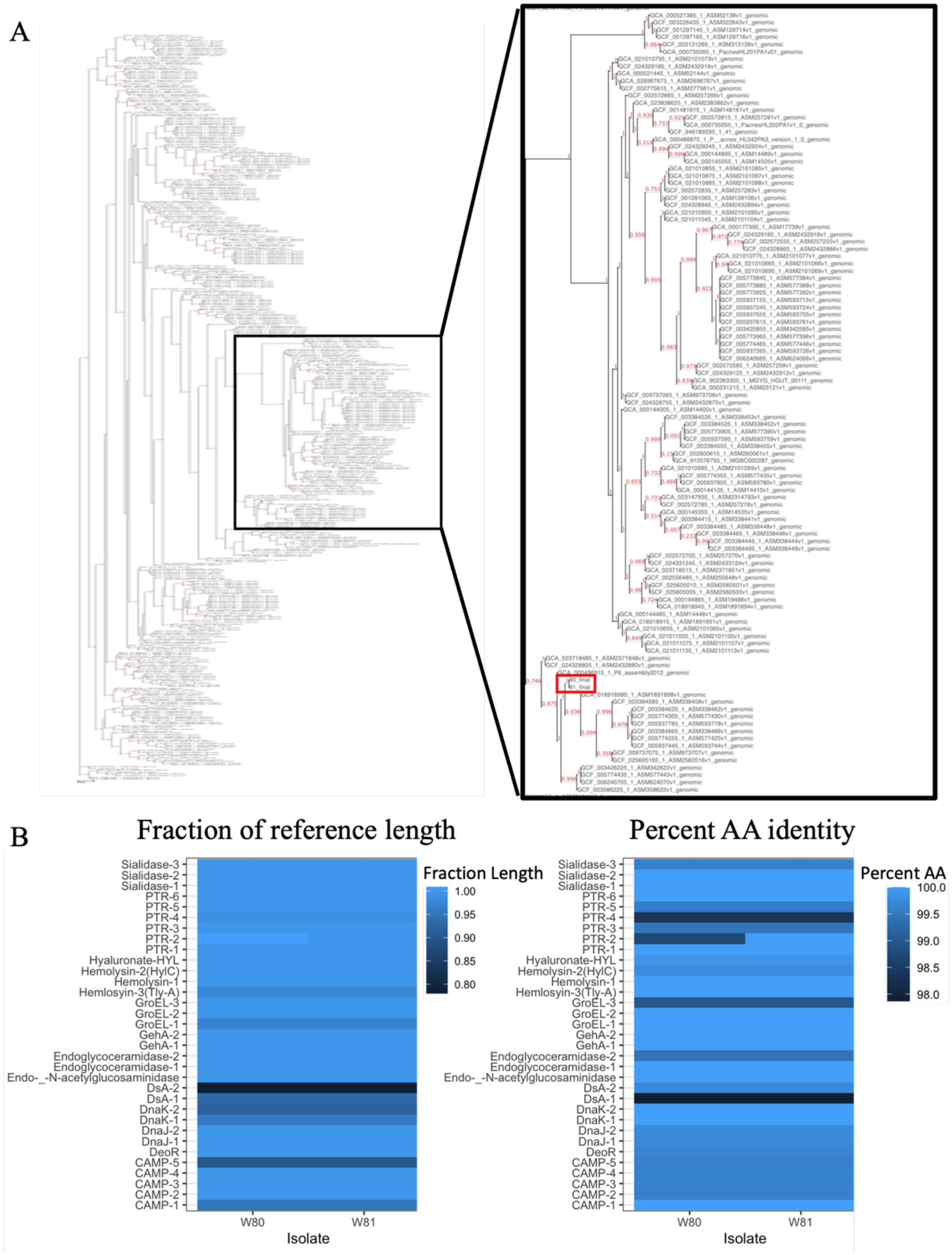

Supplementary Figure 1. Extended phylogenetic tree and comparison to KPA171202 virulence markers. (A) FastTree constructed tree of all currently available *C. acnes* genomes on NCBI. (B) Comparison of fraction of length (left) and percent amino acid identity (right) when blasted against virulence factors found in KPA171202. The newick code for the phylogenetic tree can be found at: https://github.com/LeungLab/C_Acnes

Brown DG, Wahlig TA, Ma A, Certain LK, Chalmers PN, Fisher MA, Leung DT. Genomic characterization of two *Cutibacterium acnes* isolates from a surgical site infection reveals large genomic inversion. *Pathogens and Immunity*. 2023;8(1):64-76. doi: 10.20411/pai.v8i1.606
